# Graphical Image Region Extraction with K-Means Clustering and Watershed

**DOI:** 10.3390/jimaging8060163

**Published:** 2022-06-08

**Authors:** Sandra Jardim, João António, Carlos Mora

**Affiliations:** 1 Smart Cities Research Center, Polytechnic Institute of Tomar, 2300-313 Tomar, Portugal; carlos.mora@ipt.pt; 2 Techframe-Information Systems, SA, 2785-338 São Domingos de Rana, Portugal; joao.antonio@techframe.pt

**Keywords:** K-Means, clustering, region extraction, image segmentation, connected component analysis, watershed

## Abstract

With a wide range of applications, image segmentation is a complex and difficult preprocessing step that plays an important role in automatic visual systems, which accuracy impacts, not only on segmentation results, but directly affects the effectiveness of the follow-up tasks. Despite the many advances achieved in the last decades, image segmentation remains a challenging problem, particularly, the segmenting of color images due to the diverse inhomogeneities of color, textures and shapes present in the descriptive features of the images. In trademark graphic images segmentation, beyond these difficulties, we must also take into account the high noise and low resolution, which are often present. Trademark graphic images can also be very heterogeneous with regard to the elements that make them up, which can be overlapping and with varying lighting conditions. Due to the immense variation encountered in corporate logos and trademark graphic images, it is often difficult to select a single method for extracting relevant image regions in a way that produces satisfactory results. Many of the hybrid approaches that integrate the Watershed and K-Means algorithms involve processing very high quality and visually similar images, such as medical images, meaning that either approach can be tweaked to work on images that follow a certain pattern. Trademark images are totally different from each other and are usually fully colored. Our system solves this difficulty given it is a generalized implementation designed to work in most scenarios, through the use of customizable parameters and completely unbiased for an image type. In this paper, we propose a hybrid approach to Image Region Extraction that focuses on automated region proposal and segmentation techniques. In particular, we analyze popular techniques such as K-Means Clustering and Watershedding and their effectiveness when deployed in a hybrid environment to be applied to a highly variable dataset. The proposed system consists of a multi-stage algorithm that takes as input an RGB image and produces multiple outputs, corresponding to the extracted regions. After preprocessing steps, a K-Means function with random initial centroids and a user-defined value for *k* is executed over the RGB image, generating a gray-scale segmented image, to which a threshold method is applied to generate a binary mask, containing the necessary information to generate a distance map. Then, the Watershed function is performed over the distance map, using the markers defined by the Connected Component Analysis function that labels regions on 8-way pixel connectivity, ensuring that all regions are correctly found. Finally, individual objects are labelled for extraction through a contour method, based on border following. The achieved results show adequate region extraction capabilities when processing graphical images from different datasets, where the system correctly distinguishes the most relevant visual elements of images with minimal tweaking.

## 1. Introduction

To the human visual system, an image is not just an arbitrary set of pixels, but rather a meaningful arrangement of regions and objects. Perceiving the interesting parts of a scene is a preliminary step for recognizing, understanding and interpreting an image.

The segmentation of an image consists of subdividing the image into its constituent parts (or objects), considering certain characteristics of the image such as color, intensity, texture and text, among others. In this context, an object refers to a convex component. Segmentation can be seen as a classification problem, where the objective is to classify *N* elements in *K* regions, where k ⩽ n, such that elements in the same *K* region have properties similar to each other and distinct from the properties of elements in other regions, K ⩽ N; $\overset{i=1}{\underset{K}{⋃}}K_{i}\;=$ image and $K_{i}\cap K_{j}\;=\;\emptyset ,$ if i ≠ j. In this sense, segmentation can also be modelled as a combinatorial optimization problem, where an optimal region is sought according to a certain similarity criterion between the elements of the same region. Traditionally, image segmentation can follow two strategies: discontinuity, where the image partition is performed based on sudden intensity changes (e.g., contour detection) [1,2,3]; and similarity, where the partition is performed based on the similarity between pixels, following a certain criterion (e.g., binarization, region growth, region division and joining) [4,5].

Image segmentation is a complex and difficult preprocessing step that plays an important role in automatic visual systems. It has a wide range of applications such as biometrics [6,7,8], medical image analysis [9,10,11], disease detection and classification in cultures [12,13,14,15,16], traffic control systems [1,17,18,19,20], self driving cars [21,22,23,24], locating objects in satellite images [25], and content image retrieval systems [26,27,28,29], among others.

Many image segmentation algorithms have been proposed in the literature, from the traditional techniques, such as thresholding [30,31,32,33], edge-based segmentation [34,35], histogram-based bundling, region-based segmentation [36,37,38,39], clustering-based segmentation [40,41,42,43,44], watershed methods [45,46,47,48,49], to more advanced algorithms such as active contours [50,51,52,53], graph cuts [54,55,56,57], conditional and Markov random fields [58,59,60,61], and sparsity-based methods [62,63,64].

Given that the accuracy of segmentation not only has an impact on segmentation results, but directly affects the effectiveness of the follow-up tasks, many efforts have been made by the scientific community to develop efficient image segmentation methods and techniques. Despite the many advances achieved in the last few decades, image segmentation remains a challenging problem. Particularly, the task of segmenting color images is challenging due to the diverse inhomogeneities of color, textures and shapes present in the descriptive features of the images. In trademark graphic images segmentation, beyond these difficulties, we must also take into account the high noise and low resolution, which are often present. Trademark graphic images can also be very heterogeneous with regard to the elements that make them up (pictures, text, etc.), which can be overlapping and with varying lighting conditions. In addition, in the case of trademark graphic images, we have a large amount of images to process, due to the number of new trademarks registered daily worldwide in the range of tenths of thousands, which can compromise the use of computationally heavy image segmentation methods.

In this paper, we propose an image segmentation method based on the K-Means and Watershed algorithms. The K-Means algorithm is implemented as a means to simplify image data and propose image regions based on color differences, while the Watershed algorithm performs analysis on the resulting infographical map for extracting the proposed regions, with the help of 8-way component analysis for component separation and contour detection for the final extraction. Although there are some proposals for image segmentation based on the K-Means and Watershed algorithms [65,66,67,68], to the best of our knowledge, the approach architecture we present in this paper was not previously proposed. The developed method was applied in the segmentation of trademark graphic images, which we consider to be a challenge given their characteristics. The results obtained demonstrate the robustness and efficiency of the proposed method, so we consider that the approach presented constitutes a positive contribution in the field of image segmentation.

Images with high color variation, overlapping objects and difficult shapes are rarely documented in image segmentation approaches given that they are highly variable in terms of outcome. Dealing with so many inconsistencies in images at once can either produce unusable results or require extreme amounts of fine-tuning. With our system, the usage of prior knowledge provided by K-Means and Connected Component allows for superior control over what kind of objects the user desires to extract. Simply adjusting the cluster value gives the algorithm more or less room to identify distinct blobs, while the distance value lets us directly adjust the size and distance of each object. As such, we believe our approach offers improved robustness and ease-of-use in the scope of trademark images.

In addition to integrating the proposed system into a series of image comparison systems, our objective is to develop a model matching system capable of identifying the objects extracted by this approach in other images, as well as a review of image comparison metrics as a means of obtaining results comparable to those of graphical search engines.

The remainder of this the paper is structured as follows: Section 2 describes related work and different state-of-the-art approaches used for image segmentation, focusing on methods using the K-Means and Watershed algorithms. Section 3 describes the proposed framework and related details of the proposed work. Section 4 deals with experimentation and discussion, including a sensitivity analysis of system’s variables. Section 5 presents a comparison of the proposed approach with several algorithms proposed by other authors. This section also presents the main differences between the proposed approach and those based on deep learning. Section 6 provides brief concluding remarks with conclusions and proposed future work.

## 2. Related Work

In the early 20th century, the clustering quality of the human visual system was extensively studied by psychologists following the Gestalt school [69], who identified several factors related to the human visual perception of clustering: similarity, proximity, continuity, symmetry, parallelism, the need for closure, and familiarity. In the scope of computer vision, such factors have been used as a guide for the study of many clustering algorithms and, in particular, for the investigation of the image segmentation problem [69]. The use of clustering algorithms for image segmentation stands out among the approaches available in current literature. The grouping of the characteristics of an image in the space of features implies obtaining regions in the space of the image, for which the clustering algorithms are classified as methods based on regions. In clustering methods applied to image segmentation, pixels are grouped according to similarity regarding color, texture and luminosity attributes.

### 2.1. K-Means Segmentation

Among the several clustering algorithms possible to use in image segmentation, K-Means is one of the most popular, given its simplicity and computational speed, an important feature when there are large amounts of images to be processed, and capacity to deal with a large number of variables. In this section, we discuss some of the most recent works where image segmentation follows an approach based on the K-Means algorithm.

To non-destructively detect defects in thermal images of industrial materials, Risheh et al. [70] proposes a method based on segmentation of images generated by enhanced truncated correlation photothermal coherence tomography (eTC-PCT), combined with a computer vision algorithm. The filtered eTC-PCT reconstructed image is segmented using the K-Means algorithm, and the result is applied to the delineation of the discontinuity limits using the Canny edge detection algorithm. The results presented show that the combination of these algorithms is optimal to achieve significant enhancement in the delineation of blind holes and crack contours in industrial materials.

In order to improve the reusability of part structural mesh modules, Lian et al. [71] propose a structural mesh segmentation algorithm based on K-Means clustering, where the K value is set as a controllable variable. The experimental results presented show good segmentation and friendly real-time interaction.

Nasor et al. [72] propose a fully automated machine vision technique for the detection and segmentation of mesenteric cysts in computed tomography images of the abdominal space that combines multiple K-Means clustering and iterative Gaussian filtering. Regardless of the mesenteric cysts texture variation and location with respect to other surrounding abdominal organs, the results presented show that the proposed technique is able to detect and segment mesenteric cysts, achieving high levels of recision, recall, specificity, dice score coefficient and accuracy, indicating a very high segmentation accuracy.

Zheng et al. [40] propose an adaptive K-Means image segmentation method that starts by transforming the color space of images into LAB color space, after which the relationship between the K values and the number of connected domains is used to adaptively segment the image. The authors conclude that the proposed method achieves accurate segmentation results with simple operation and avoids the interactive input of K value.

To classify the quality of Areca nut, Patil et al. [73] propose a method where the nut boundary is detected using K-Means segmentation, followed by Canny edge detection. When compared with eight different techniques of image preprocessing, the authors conclude that K-Means segmentation achieves one of the three best results for applications involving Areca nut segregation.

### 2.2. Watershed Segmentation

An investigation of the utility of a two-dimensional Watershed algorithm for identifying the cartilage surface in computed tomography (CT) arthrograms of the knee up to 33 min after an intraarticular iohexol injection is made in [74]. The proposed approach shows that the use of watershed dam lines to guide cartilage segmentation shows promise for identifying cartilage boundaries from CT arthrograms in areas where soft tissues are in direct contact with each other.

Hajdowska et al. [57] propose a method that combines Graph Cut, Watershed segmentation and Hough Circular Transform to improve automatic segmentation and counting living cells, to overcome image segmentation difficulties on top-down pictures with overlapping cells.

Banerjee et al. [75] propose a method where Watershed segmentation is used to cross-validate the classification of lung cancer.

To tackle the problem of identifying authentic and tampered images created by the copy-move forgery technique, Dixit et al. [76] propose a method in which Stationary Wavelet Transform (SWT) and spatial-constrained edge preserving Watershed segmentation are applied over input images in preprocessing steps. The results obtained show that the proposed approach can effectively distinguish between forged and original images containing similar appearing but authentic objects, being also able to detect forged images sustaining different post-processing attacks.

Shen et al. [77] propose an adaptive morphological snake based on marked Watershed algorithm for breast ultrasound image segmentation, where the candidate contours of the marked areas are obtained with a marked watershed. The results presented show that the approach is robust, efficient, effective and more sensitive to malignant lesions than benign lesions.

Hu et al. [78] propose a text line segmentation method based on local baselines and connected component allocation, where the Watershed algorithm is used to segment touching connected components. The results presented show that the proposed approach can effectively reduce the influence of text line distortion and skew on text line segmentation, presenting a high degree of robustness, and a good segmentation accuracy for image text lines in Tibetan documents with touching and broken strokes.

Tian et al. [46] proposed a successful approach based on Watershed segmentation to build automatic citrus decay detection models in image-level.

### 2.3. Hybrid Segmentation

In literature, we can find some proposals for hybrid approaches, where the K-Means and Watershed algorithms are used simultaneously for image segmentation.

Desai et al. [66] propose an automatic Computer-Aided Detection for early diagnosis, and classification of lung cancerous abnormalities, where the segmentation process is performed through Marker-controlled watershed segmentation and the K-Means algorithm.

An improved hybrid approach to detect brain tumor is proposed by Tejas et al. [65] by combining sequentially watershed segmentation, K-Means clustering and level set segmentation. When compared with other techniques like Threshold segmentation, K-Means clustering, Watershed segmentation and Level set segmentation, the proposed hybrid approach show a better specificity, accuracy, and precision, and more precise tumors detection.

## 3. Methods

As previously stated, image segmentation is a complex and difficult task, where its accuracy not only has an impact on segmentation results, but directly affects the effectiveness of the follow-up tasks. It plays an important role in automatic visual systems, with a wide range of applications. In this paper, we propose a hybrid algorithm for segmentation and extraction of graphical objects in trademark images. The focus on this type of images comes from the fact that the proposed method will integrate a trademark content-based image retrieval system, to be used in the intellectual property surveillance.

### 3.1. System Description

The proposed system consists of a multi-stage algorithm that takes a single input image and produces multiple outputs. Given any RGB image, the standard procedure is as follows:

Step 1: Preprocessing steps resize the input image to size 224 × 224 and reshape image information into a three-dimensional array. When the image is first read, it is saved as a one-dimensional numerical array containing information relative to each and every pixel. Because we are going to work with RGB images, reshaping this information into three dimensions assures one channel is used for each of the three red, green and blue color ways.
Step 2: A K-Means function with random initial centroids and a user-defined value for *k* is executed. This step performs initial segmentation in the input image and removes noise caused due to inferior image quality. In typical implementations, using randomized cluster centroids may negatively impact the clustering result. However, in this scenario, K = 2 is persistently used as a default value, given that the main objective of this algorithm is to separate foreground from background, where randomized cluster centroids should not produce adverse results.
Step 3: A gray-scale version of the K-Means segmented image is generated to be input into a threshold function. Thresholding is a method in which each pixel of a certain image is replaced by a black pixel, given that the image intensity $I_{i}{,}_{j}$ is lower than a user-defined fixed constant, *T*, or a white pixel, given that the image intensity is higher than the constant [79]. For the proposed system, a constant *T* value of 225 was used, due to the K-Means implementation already performing basic segmentation of image components by creating two distinct color labels and thus eliminating the need for a lower (generally 128) *T* value. The goal of this threshold method is to generate a binary mask containing information necessary for distance mapping.
Step 4: The binary mask produced in step 3 contains information regarding foreground and background labeled-pixels and is then used to generate a distance map calculated through the exact distance transform formula. The exact distance transform computes the distance from non-zero (foreground) points to the nearest zero (background) points and allows for binary input [80]. The distance map is an input step necessary for Watershed functions as it labels each pixel with the distance to the nearest obstacle pixel (in this case, another object boundary). Connected Component Analysis is then performed over the distance map, labelling regions based on 8-way pixel connectivity and generating markers that ensure all regions are correctly found.
Step 5: The Watershed function is performed over the distance map with the mask generated in step 3, and using the markers defined by the CCA function as described in the scikit image documentation [81]. This step unifies the processes performed in earlier steps and aims to separate any overlapping objects. Finally, individual objects are labelled for extraction via the contours method. The minimum distance to consider for each local maximum can be set by a user-defined parameter.
Step 6: Because the output of the previous watershed step is a binary image, using an edge detection system like Canny is not necessary for Contour detection. The proposed method uses a border proposed by Satoshi Suzuki [82] for finding extreme outer contours through the labels created in the previous step, outlining each separate object and extracting it into a separate file. Each of the outputs is presented as a binary mask.

**Figure 1:**
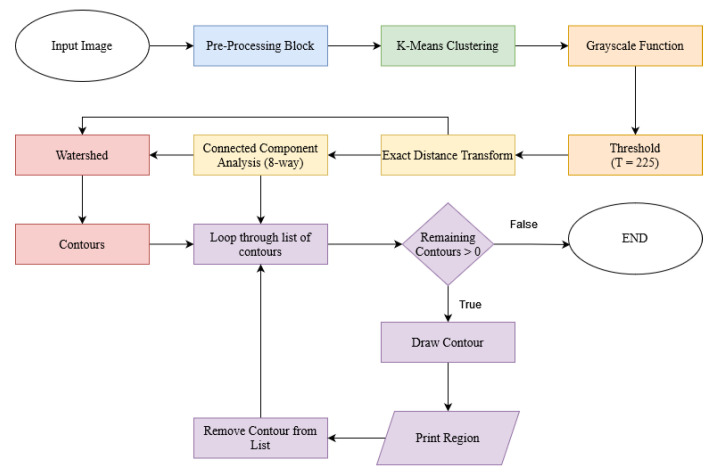
Proposed approach block diagram.

Overall, the proposed method aims to deliver a linear solution capable of processing graphical images with low visual complexity, as the main goal of this method is to be implemented in an brand image processing system, where simplistic logos and emphasis on graphical elements are the predominant features found. As a means to provide visual aid to the typical algorithm function cycle, we present a proposed approach block-diagram in Figure 1.

### 3.2. Time Complexity of the Proposed Approach

In order to define the time complexity of the multi-stage algorithm proposed, it is necessary to define the time complexity of each of its steps. The preprocessing techniques performed in step 1 have a linear complexity of O(n), where *n* is the number of image pixels. The complexity of K-Means is O(n × k × m × i) [83], where *k* is the number of clusters to consider, to which corresponds the number of colors in the output image, *m* is the dataset dimensionality, that is, the number of features, and *i* the number of iterations needed to achieve convergence. Given that the number of clusters required is much lesser than the data size, k << n, and that the data are not dimensionally big (m = 3), step 2 of the proposed approach takes the linear time complexity O(n) to label all the image pixels. Step 3 performs an RGB to gray-scale image transform, which has a linear complexity, followed by a threshold function, whose complexity is O(L × n), with *L* the number of pixel intensities. With the threshold function applied to an 8-bit grayscale image, we have L = 256. Thus, step 3 has a time complexity of O(n). Step 4 begins by generating a distance map from the binary mask produced in step 3, which has a linear complexity of O(n) [84]. CCA has a complexity of O(d × n), for a *d*-way connectivity [85,86]. In the proposed approach, the markers are generated considering an eight-connectivity. The marker-controlled Watershed function, performed in step 5, has a complexity of O(n) [87,88,89]. In the last step of the proposed approach (step 6), a contour detection method with a time complexity of O(n) is performed, bringing the overall time complexity of the proposed approach to O(n) [82].

Although the time complexity of the proposed approach is as linear as O(n), where *n* is the number of image elements, the system processing speed is heavily influenced by the used data structures, software optimizations, algorithms implementation, among other factors [90]. In addition to the computational complexity, it is important to estimate the overall number of operations of the proposed system, given its impact on its processing speed. Given the block-diagram of the proposed approach (Figure 1) and assuming that each elementary operation is counted as one floating point operation (flop), the estimated number of operations performed by each block is the following:

- Preprocessing: The resizing of the image to be processed and its representation in a three-dimensional RGB array have *n* operations each.
- K-Means: Considering the sequential implementation of the K-Means algorithm, the amount of computation within each K-Means iteration is constant. Each iteration consists of distance calculations and centroid updates. Distance calculations require roughly (3nkm + nk + nm), where 3nkm is the number of operation needed to compute the squared Euclidean distance, nk is the number of operations needed to find the closest centroid for each data point, and nm is the number of operations needed for the reassignment of each data point to the cluster whose centroid is closest to it. Centroid updates require approximately km operations. Hence, the estimated number of operations performed by the sequential implementation of the K-Means algorithm can be estimated as (3nkm + nk + nm + km) × i.
- Grayscale function and Threshold: Each block requires *n* operations.
- Exact Distance Transform: The proposed approach calculates the distance map using the function provided by the OpenCV library, which implements the algorithm presented by [84], whose number of operations is 2n.
- Connected Component Analysis: Performed using the two-pass algorithm implementation proposed by [85], based on the Rosenfeld algorithm [86], where the number of operations performed in each scan of the image is $2N_{G}n$, with $N_{G}\;=\;8$, for 8-connectivity neighbourhood.
- Watershed: The Watershed function is performed over the previously generated distance map and using the markers defined by the CCA function, according to the algorithm proposed by Beucher and Meyer [88]. Considering a $N_{G}n$-connectivity, where $N_{G}\;=\;8$, and the implementation proposed by Bieniek and Moga [89], the estimated number of operations required for the execution of the Watershed algorithm is $4N_{G}n$.
- Contour Detection: Performed through the implementation of the algorithm proposed by Satoshi Suzuki [82], requires $N_{G}n$ operations, with $N_{G}\;=\;8$, for 8-connectivity neighbourhood.

Hence, the estimated overall operation number of the proposed approach is 46 *n* + (10 *kn* + 3 *n* + 3 *k*) *i* $C_{1}n\;+\;\left(C_{2}\right)kn\;+\;C_{3}\left(k\;+\;n\right))\;\times \;i$ floating point operations, with $N_{G}\;=\;8$, m = 3, and i ≤ 100.

### 3.3. K-Means Clustering

The clustering analysis algorithm divides the data sets into different groups according to a certain standard, so it has a wide application in the field of image segmentation.

K-Means is a clustering function that aims to partition a set of observations ($x_{1},\;x_{2},\;\ldots ,\;x_{n}$), where each observation is a d-dimensional real vector, into k(≤n) sets $S\;=\;(S_{1},\;S_{2},\;\ldots ,\;S_{k})$ so as to minimize the within-cluster sum of squares. The most common implementation of the K-Means algorithm, and the one used throughout this experiment, uses an iterative refinement technique. Given an initial set of *k* means $m_{1}^{\left(1\right)},\;\ldots ,\;m_{k}^{\left(1\right)}$, the algorithm functions by alternating between the assignment step and the update step. The assignment step aims to assign each observation to the cluster with the nearest mean:

(1) $$S_{i}^{\left(t\right)}=\left\{x_{p}:∥x_{p}-\mu_{i}^{\left(t\right)}{∥}^{2}\leq ∥x_{p}-\mu_{j}^{\left(t\right)}{∥}^{2}\;,\forall j,1\leq j\leq k\right\},$$

After assigning an observation, the update step recalculates means (centroids) for observations assigned to each cluster:

(2) $$\mu_{i}^{\left(t+1\right)}=\frac{1}{|S_{i}^{\left(t\right)}|}\sum_{x_{j}\in S_{i}^{\left(t\right)}}x_{j},$$

**Figure 2:**
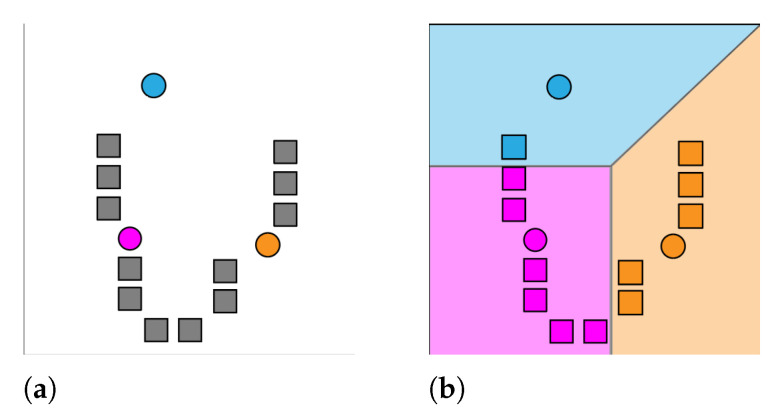
K-Means assignment step. (**a**) *k* initial means are randomly generated, k = 3; (**b**) *k* clusters are created by associating observations with the nearest mean clustering.

**Figure 3:**
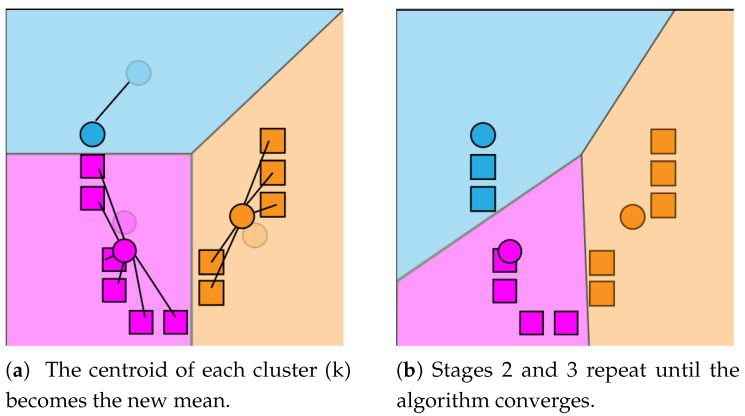
K-Means update step (**a**) and loop (**b**).

The K-Means algorithm will continue alternating until it converges, meaning the assignments are no longer changing with each iteration. It is not guaranteed that the algorithm will find the optimum. See Figure 2 and Figure 3 for demonstration of the K-Means functionality. The K-Means clustering function can be applied to image data as a means to reduce the quantity of information, while still providing an accurate representation of the image contents. More often than not, unprocessed color image files contain noise around image edges, mostly due to compression techniques. This noise is usually unnoticeable until the image is thresholded as to only display pure black or white pixels. By clustering similarly colored pixels together, noise of this nature is completely removed as it is merged with background pixels.

### 3.4. Watershed Algorithm

The watershed algorithm is mainly used for segmentation within an image, that is, for separating objects in an image [91]. This approach is mainly used for separating two or more overlapped objects due to the way it operates. Given a gray scale image, the watershed technique consists of treating pixel values as a local topography map, the brighter the pixel, the bigger its height. Once every pixel has a height value, the algorithm proceeds by simulating flooding, starting at the basins (lower points) and stopping once the lines that run along the tops of ridges are found [92]. From this point on, an estimated distance between the middle-points of both objects is calculated and the objects are separated. The distances between peaks are calculated using the two-dimensional Euclidean distance formula. In the Euclidean plane, let point *p* have Cartesian coordinates (p1,p2) and let point *q* have coordinates (q1,q2). Then, the distance between *p* and *q* is given by [93]:

(3) $$d\left(p,q\right)=\sqrt{{\left(q_{1}-p_{1}\right)}^{2}+{\left(q_{2}-p_{2}\right)}^{2}}$$

**Figure 4:**
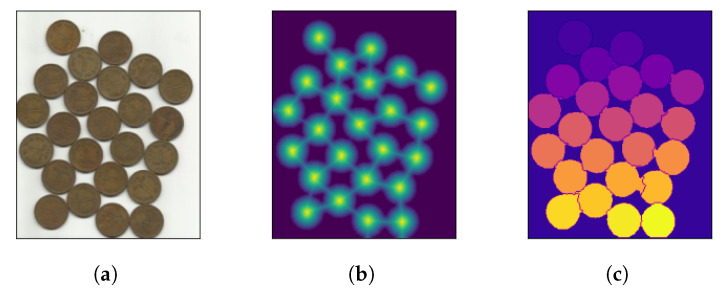
Watershed algorithm functionality in three steps. (**a**) overlapping objects; (**b**) distances; (**c**) segmented objects.

After the Euclidean distance between peaks is computed, the distance map can be worked upon to find the local maxima of the image. It is possible to set a minimum distance to consider between each peak, as this is important when processing images with very densely packed objects. Upon finding all peaks, most watershed implementations resort to an eight-way connected component analysis for finding objects defined by connected pixels. Figure 4 demonstrates how a typical watershed algorithm operates in three steps.

### 3.5. Connected Component Analysis

**Figure 5:**
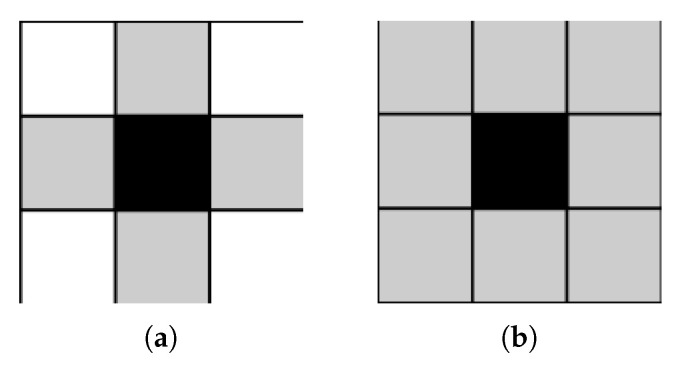
CCA with 4-way and 8-way connectivity. (**a**) 4-way connectivity; (**b**) 8-way connectivity.

In addition, referred to as Connected Component Labelling, CCA is an algorithmic application of graph theory that focuses on subsets comprised of connected pixels. It is widely used in computer vision to detect connected regions in binary digital images. It operates upon image information, constructing a graph containing vertices and connected edges. A typical CCA algorithm traverses the graph, labelling vertices based on their connectivity with surrounding neighbours. Graphs are usually 4-connected (Figure 5a) or 8-connected (Figure 5b) [94]. Four-connected pixels are neighbours to every pixel that touches one of their edges, either horizontally or vertically. Coordinately, every pixel containing coordinates (x ± 1,y) or (x,y ± 1) is connected to the pixel at (x,y). Eight-connected pixels are neighbours to every pixel that touches one of their edges or corners, vertically, horizontally or diagonally. Apart from 4-connected pixels, any pixel with coordinates (x ± 1,y ± 1) is connected to the pixel at (x,y).

### 3.6. Contour Detection

**Figure 6:**
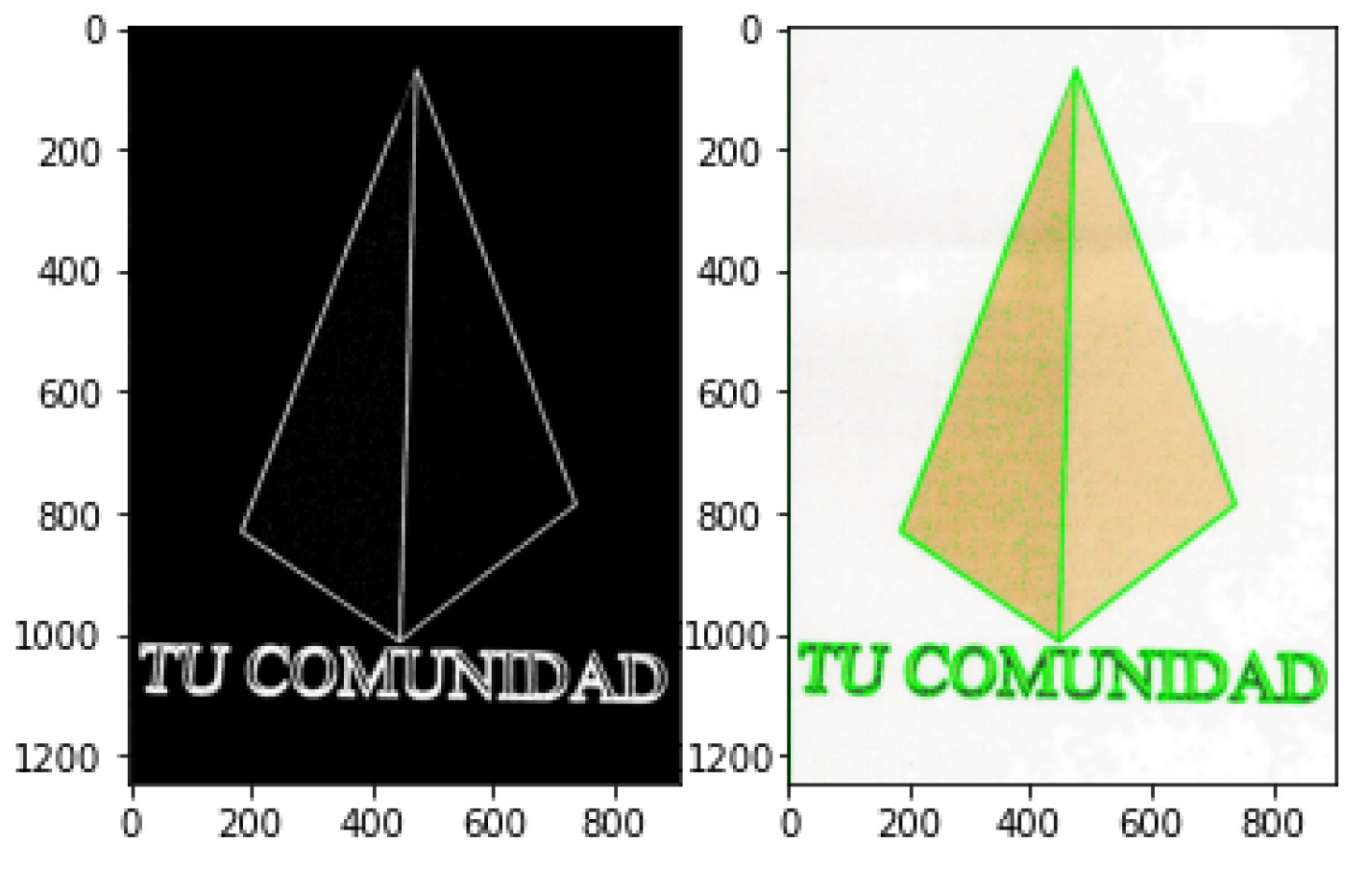
Contour detection in objects.

**Figure 7:**
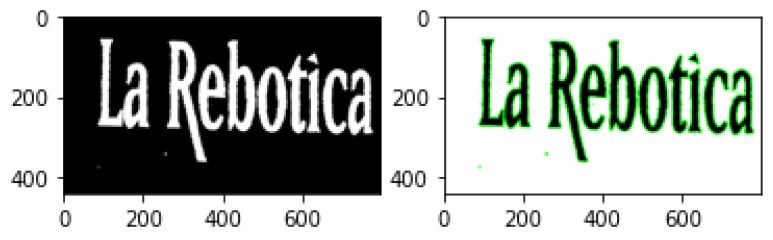
Contour detection in text.

Contour Detection is an application of edge detection for outlining thresholded images by detecting color changes and marking them as contours. Contour detection is widely used in motion detection systems or segmentation mechanisms. A typical contour detection algorithm reads an input image in gray-scale format before applying a threshold method, converting the image to pure black and white. Finding contours involves comparing pixels with their neighbours and detection any color changes. Because every pixel is either black (0) or white (255), the difference is easily noticeable. Contour locations are recorded and drawn over the original RGB image. This technique can be useful not only for highlighting objects in an image (Figure 6), where an obvious difference in pixel intensity is tagged as a contour, but it can also be exceptionally functional when highlighting text (Figure 7), since fonts are usually properly spaced out and allow for easy detection. The following examples were produced with the same extreme outer contour mechanism used in the proposed approach and described in the opencv documentation [82].

## 4. Results

This section describes the experimental results when processing a portion of the dataset mentioned in Section 4.1. Although the used dataset is not publicly available as a whole, it is entirely comprised of public images and can be reproduced. Overall, the system we presented correctly separates recognizable shapes and objects in most given images. For evaluation and testing purposes, we input images of variable visual complexity into the system. Table 1 shows the average processing times needed for extracting regions out of the datasets images using the proposed system. This algorithm execution was performed on a remote machine offered by Google Colab’s services, running Python 3.6.9 and containing a Tesla K80 graphics card paired with 12 GB of RAM.

During the following experiments, textual features found in images are usually disregarded completely by the algorithm. This occurs during the watershed step, as the minimum distance defined to be considered between each local maxima tends to be higher than the character spacing used in most fonts that are not heavily stylized or large in size. Most text found in images does not provide relevant information unless semantically analysed, so this does not constitute a fault in the algorithm.

### 4.1. Dataset

The aforementioned system was tested on two datasets comprised of images of variable size, content and complexity, representing trademark images. Trademark images are aggregated in sets corresponding to different jurisdictions, namely international, national and regional, which together constitute the totality of trademark images registered in the world. The images used belong to two datasets of different scopes, one corresponding to a national jurisdiction and the other to an international jurisdiction. The morphologies of the two datasets are considerably different, the first corresponding to more complex images with more textual elements, mostly in one language (Portuguese) and the second dataset containing simpler images, with faster visual identification, with fewer textual elements and a large diversity of languages. Dataset 1 is comprised of 19,102 images of varied jurisdictions and dataset 2 contains 45,690 images registered solely on the Portuguese jurisdiction.

### 4.2. Experimental Results

Next, the results obtained for a set of images from the two previously mentioned datasets are presented and analysed. For each input image, the output images of the main blocks of the proposed approach are presented.

#### 4.2.1. Experiment A

**Figure 8:**
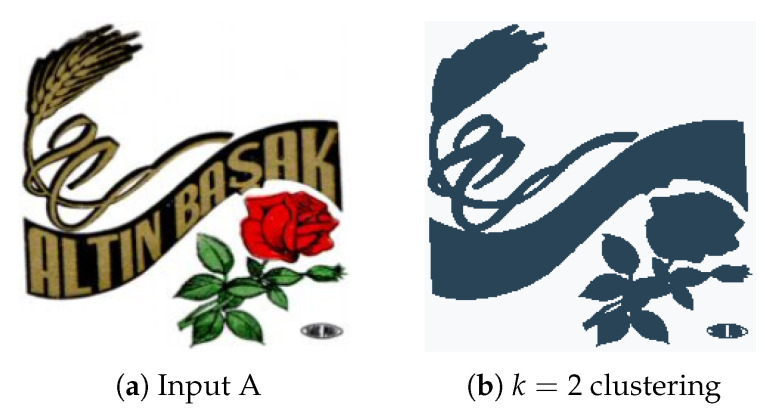
(**a**) Experiment A input; (**b**) Experiment A K-Means clustering (*k* = 2).

**Figure 9:**
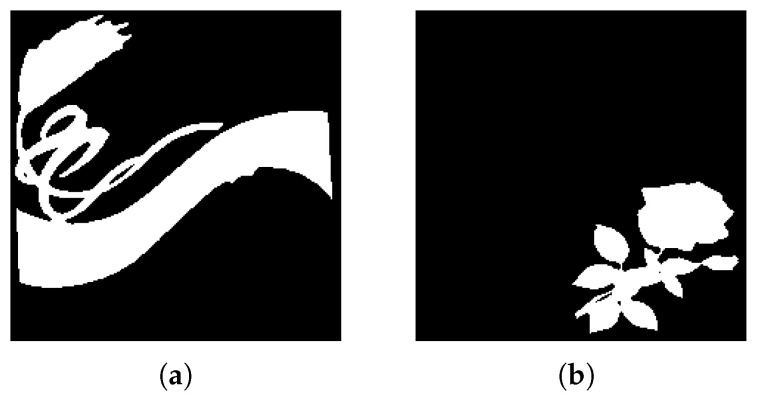
Experiment A extracted regions (**a**) First extracted region; (**b**) Second extracted region.

Experiment A consists of the benchmark test carried out for system performance evaluation. Initially, the input image (Figure 8a) was simplified through the use of K-Means clustering with k = 2, resulting in a binary (though not black and white) image. With the K-Means labels used as reference for the watershed and CCA techniques, and considering the minimum distance between the edges of objects d = 30, each distinct region is processed and tagged for the contour detection algorithm to identify and extract. In this scenario, both major regions of the input image were correctly extracted, although a separation between the upper two objects in output Figure 9a would be ideal. We believe that the small artifact in the bottom-right corner of the original image was not properly labelled during the watershed function and therefore was lost in the contour drawing process.

#### 4.2.2. Experiment B

**Figure 10:**
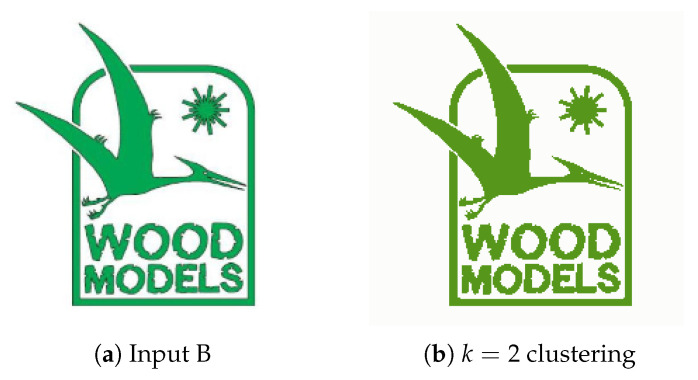
(**a**) Experiment B input; (**b**) Experiment B K-Means clustering (*k* = 2).

**Figure 11:**
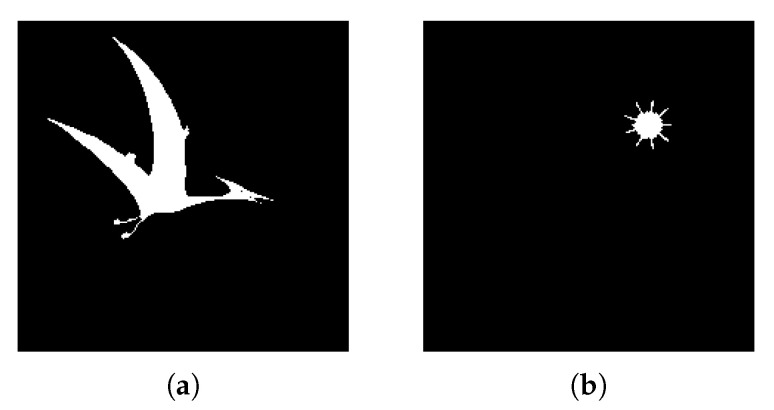
Experiment B extracted regions (**a**) Object A; (**b**) Object B.

Experiment B, with the input image presented in Figure 10a aims to represent the usage of this system for removing unnecessary visual elements from images, such as text, frames and complementary visual components. In image segmentation implementations, text is often regarded as noise due to the little information it provides as an object. Figure 11a displays the Watershed function’s ability to separate overlapping objects, the underlying frame shape receives a label different to that of the object in front and, as such, CCA and Contour Detection successfully extract the more meaningful object. Since the distance between the letters is less than 30, taken as a minimum distance between the edges of objects d = 30 is possible to disregard the text present in the image. This value of *d* also allows not extracting the image frame, which is a meaningless object given the purpose of the developed system.

#### 4.2.3. Experiment C

**Figure 12:**
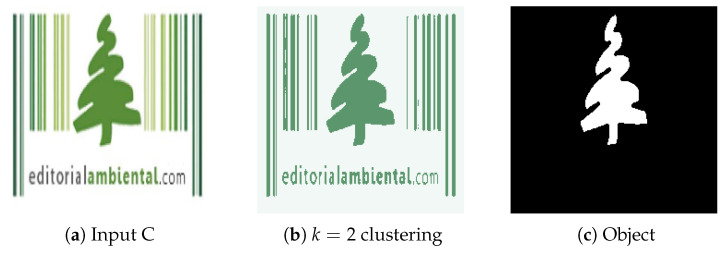
(**a**) Experiment C input; (**b**) Experiment C K-Means clustering (*k* = 2); (**c**) Experiment C extracted region.

Experiment C also displays the object isolating capabilities of the system. Figure 12a contains a single main object and other minor, surrounding visual elements. By setting the adequate minimum distance to consider between objects (d = 30), the watershed function effectively removes unnecessary artifacts such as vertical lines and text because they usually are either grouped together (text), meaning the distance between each component is very low, or are simply not dense enough to constitute a meaningful object (lines). In this case, a higher minimum distance (d = 20) has been defined and the algorithm correctly separates a single region from the image.

#### 4.2.4. Experiment D

**Figure 13:**
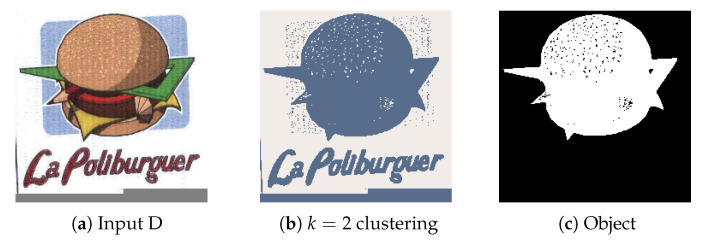
(**a**) Experiment D input; (**b**) Experiment D K-Means clustering (*k* = 2); (**c**) Experiment D extracted region.

Experiment D aims to represent the noise removal capabilities of the techniques utilized. The input image (Figure 13a) contains noise that could not be completely removed during the clustering phase (Figure 13b). However, consider that only objects formed by grouped up pixels (Connected Component Analysis) deal with most of the static found around the central object, while watershedding with proper distance values eliminates other features such as text and meaningless objects.

#### 4.2.5. Experiment E

**Figure 14:**
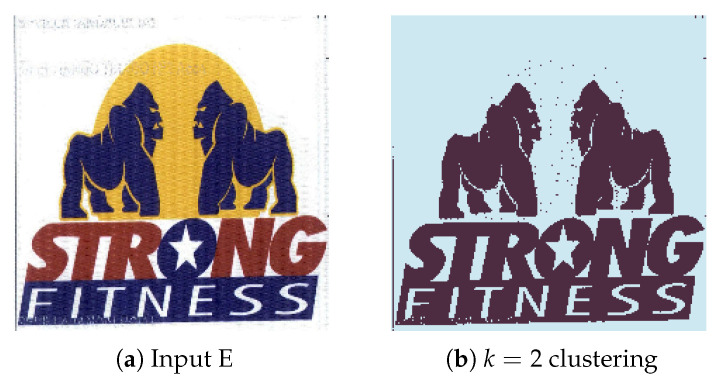
(**a**) Experiment E input; (**b**) Experiment E K-Means clustering (*k* = 2).

**Figure 15:**
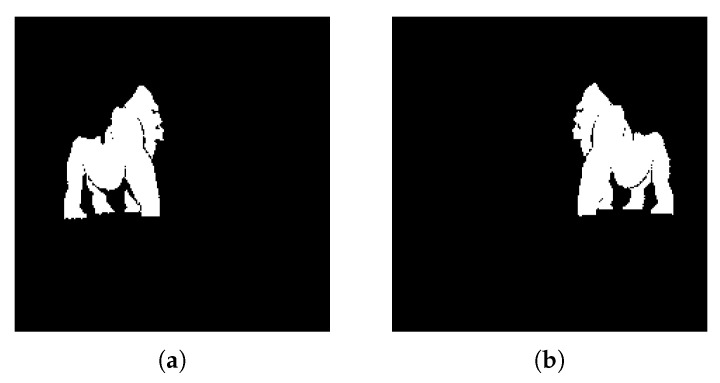
Experiment E extracted regions (**a**) Region A; (**b**) Region B.

The current experiment aims to demonstrate the algorithm functionality in a more visually dense environment. The two objects we aim to extract from the image in Figure 14a are in front of a colored background and there are stylized font elements that can create a difficult extraction scenario. By tweaking the watershed distance value between local maxima (d = 25), we managed to obtain a clean detection and extraction of both animals in outputs Figure 15a,b. In this scenario, we observe the K-Means function’s use of simplifying image information, where the yellow object behind the two significant objects was merged into the background and therefore eliminated, allowing for easier background and foreground labelling.

#### 4.2.6. Experiment F

**Figure 16:**
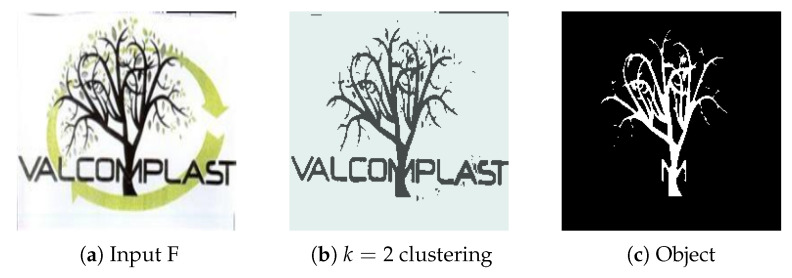
(**a**) Experiment F input; (**b**) Experiment F K-Means clustering (*k* = 2); (**c**) Experiment F extracted region for d = 25.

We chose contender image Figure 16a as a relevant test due to the visual complexity of the object we wish to extract. There are many tree branches with underlying elements which can cause unsatisfactory extraction if not properly identified, such as an incomplete tree or background elements bleeding onto the proposed region.

The result is satisfactory, with detailed branches and a degree of elimination for smaller objects (leaves). However, as expected, the character “M” was carried over to the extracted object.

#### 4.2.7. Experiment G

**Figure 17:**
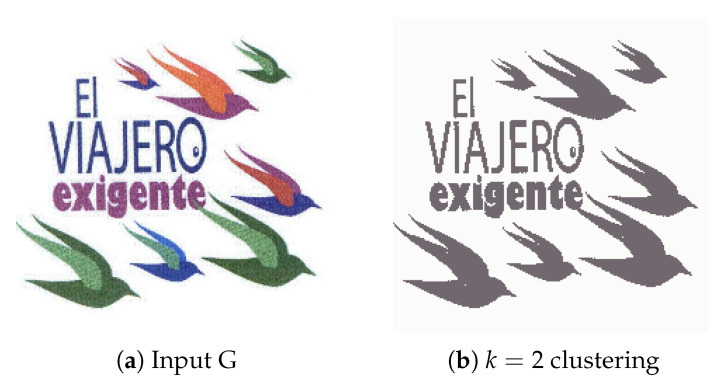
(**a**) Experiment G input; (**b**) Experiment G K-Means clustering (*k* = 2).

**Figure 18:**
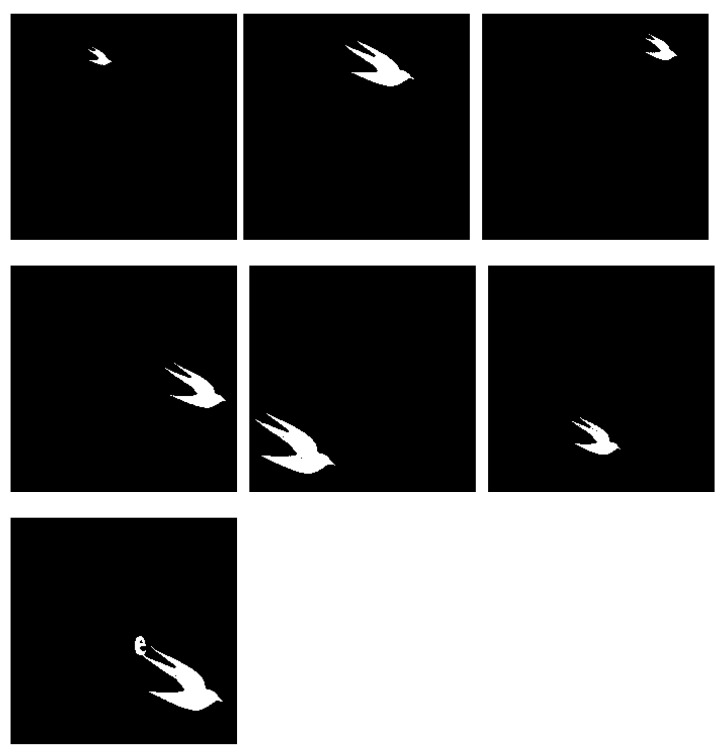
Experiment G extracted regions for d = 25.

Lastly, Figure 17a presents an image with a big number of distinct, well-separated regions of variable sizes and evaluated how the system performs in identifying them. We expect each individual bird to be proposed as an individual region with no text elements in the outputs. The results presented in Figure 18 show good accuracy in capturing most birds, but due to connected component labelling, one of the outputs is attached with the letter ‘e’. This unfortunately could not be solved by adjusting watershed distances or increasing the number of clusters, and as such, the difficulty associated with images containing attached visual elements requires some refinement.

### 4.3. System Variables Sensitivity

Sensitivity analyses involve varying system inputs to assess the individual impacts of each variable on the output and ultimately provide information regarding the different effects of each tested variable. The developed system has an RGB image as input, producing from 1 to *p* outputs, depending on the number of objects extracted from the processed image. The system variables are the number of clusters in the K-Means algorithm (*k*) and the minimum distance to consider between the edges of objects (*d*). The threshold value is not considered a system variable since the value remains fixed to T = 225, as previously stated. To evaluate system variables sensitivity, a set of experiments were performed on several input images using different values for the variables *k* and *d*.

For images where the objects are inherently separated from each other, it is verified that the system performs as expected, extracting the same objects for different values of *k*, with k ≥ 2, which show its stability for different considered clusters. In the presence of overlapping objects, the value of *k* must be greater than 2, allowing complete extraction of objects at the top level. From the results obtained, it is possible to verify that, also in these cases, the system remains stable for different values of *k*, with k ≥ 3.

Regarding the variable *d*, it can be seen that, as its value increases, less separate objects are extracted. Although this represents that the system is sensitive to the *d* variable, this is intended, giving the user more freedom for adjusting the system to extract the most relevant objects. In the proposed approach, and taking into account the objective for which it is intended, the value of *d* that is considered adequate is the one that allows only the extractions of relevant graphic objects, disregarding non relevant information, such as text.

**Figure 19:**
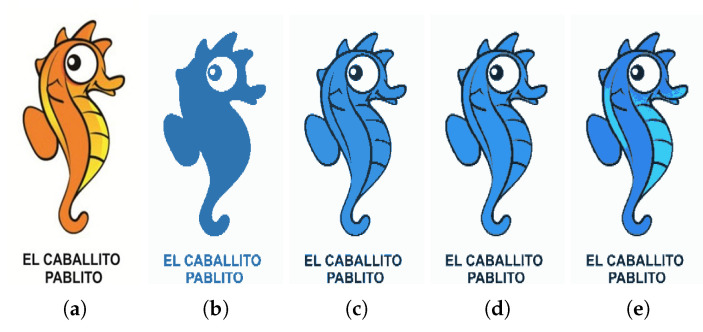
(**a**) Input image; (**b**) k = 2 clustering; (**c**) k = 3 clustering; (**d**) k = 4 clustering; (**e**) k = 5 clustering.

Figure 19 shows the segmented images resulting from K-Means clustering of the input image Figure 19a for k∈{2,3,4,5}. Since the image is formed only by a graphic object, its segmentation from the background is correctly performed by the K-Means algorithm, performed with k=2.

**Figure 20:**
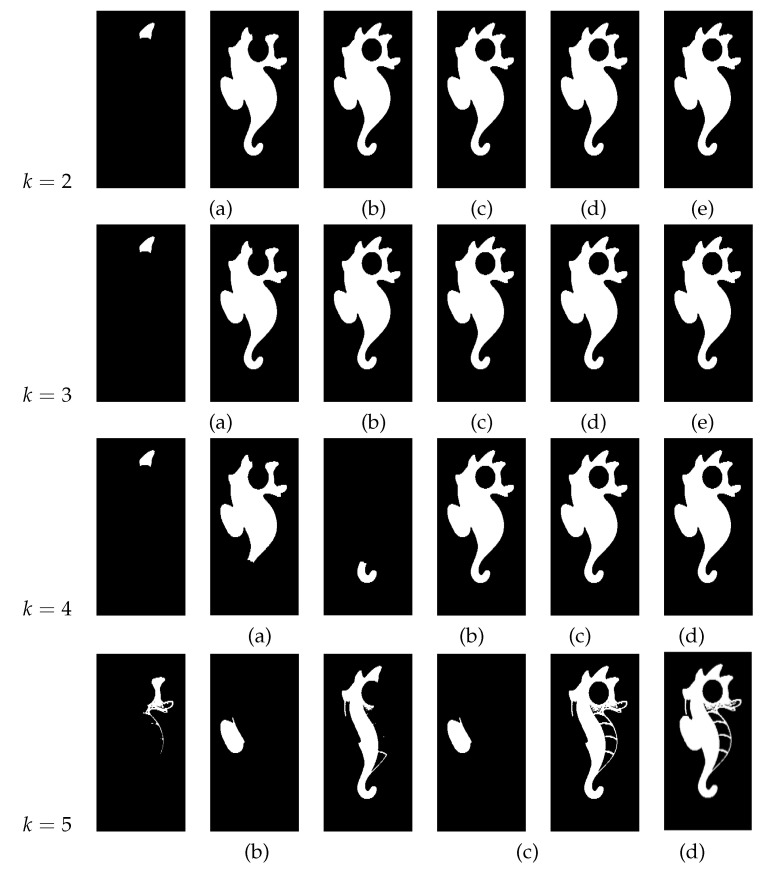
Extracted regions for different values of *d* (**a**) d=25; (**b**) d=30; (**c**) d=40; (**d**) d=45; (**e**) d=50.

Figure 20 shows, for each value of *k*, the extracted regions from the input image Figure 19a, for different values of *d*. From the results, it is possible to see that, for k∈{2,3,4}, the proposed approach achieves a good performance for d ≥ 30. For these values of *k*, when d = 25, more than one object is extracted, which is a consequence of the minimum distance between possible objects being greater than this value. When this happens, the Watershed algorithm considers different objects whenever the distance between two possible different objects exceeds d = 25. For k ≥ 5, the proposed approach provided extra details in the proposed regions opposed to K ≤ 4, where objects are mostly comprised of flat textures depicting their overall shape. This is due to the fact that the value of *k* has a direct impact on the connected component analysis function effectiveness. A lower value for *k* means the overall image has larger color blobs, which usually translates into more generalized connected regions, lacking detail and often representing the overall shape of the objects.

**Figure 21:**
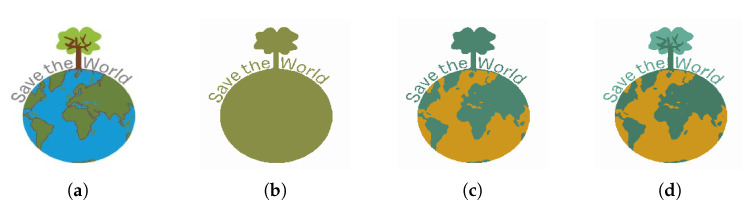
(**a**) Input image; (**b**) k = 2 clustering; (**c**) k = 3 clustering; (**d**) k = 4 clustering.

Figure 21 shows the segmented images resulting from K-Means clustering of the input image Figure 21a for k∈{2,3,4}. In this image, the graphic objects representing the continents are superimposed on the graphic representation of the oceans. The application of the K-Means algorithm with k = 2 only allows the segmentation of the circle that represents the shape of the planet Earth and the tree that emerges from its upper portion. When the K-Means algorithm with K ≥ 3 is applied to the image, it is possible to segment the regions corresponding to the continents, thus enabling their extraction.

**Figure 22:**
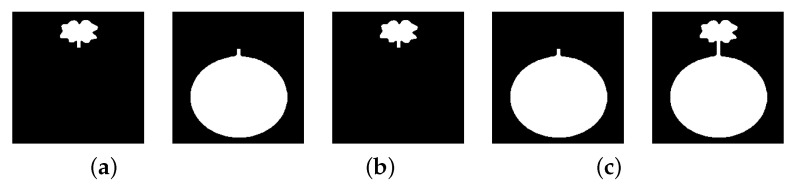
Extracted regions for k = 2 and different values of *d* (**a**) d = 25; (**b**) d = 40; (**c**) d = 50.

Figure 22 shows, for k = 2, the extracted regions from the input image Figure 21a, for different values of *d*.

**Figure 23:**
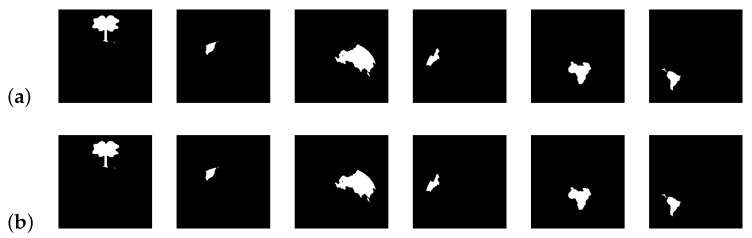
Extracted regions for d = 20 and different values of *k* (**a**) k = 3; (**b**) k = 4.

**Figure 24:**
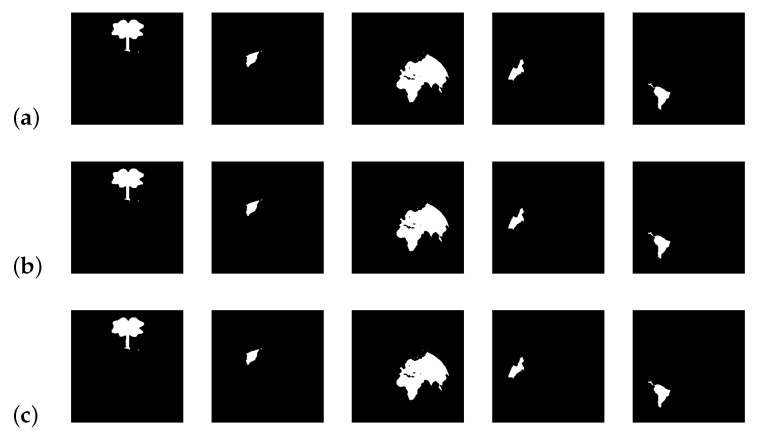
Extracted regions for different values of *k* and *d* (**a**) k = 3 and d = 30; (**b**) k = 3 and d = 40; (**c**) k = 4 and d = 30.

**Figure 25:**
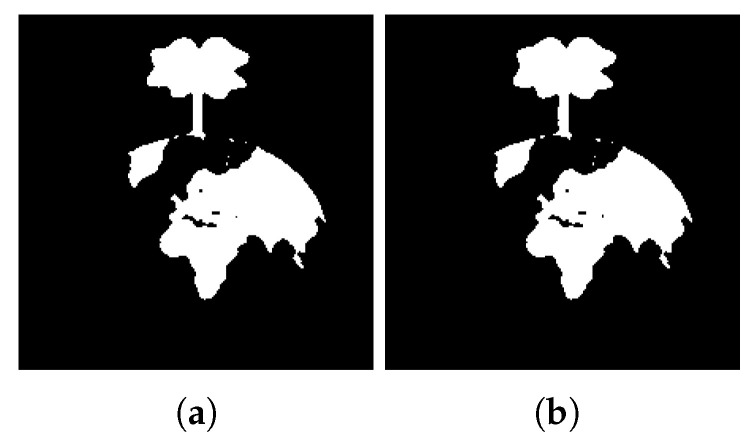
Extracted regions for d = 50 and different values of *k* (**a**) k = 3; (**b**) k = 4.

Figure 23, Figure 24 and Figure 25 show, for k∈{3,4}, the extracted regions from the input Figure 21a, for different values of *d*.

From the results, it is possible to see that, for k = 2, the extracted regions are the same for all the tested values of *d*. When k ≥ 3, and given the proximity of different regions, greater values of *d* result in the extraction of bigger regions. These results show the sensibility of the developed approach to the variable *d*, allowing an adjustment of the system regarding the details of the regions to be extracted.

## 5. Comparison of the Proposed Solution with Other Approaches

Although the proposed approach aims to extract regions of interest, more specifically relevant objects, in graphic images, its performance was evaluated by comparing its results in natural images. Natural images depict real life objects and subjects, usually presenting textures, smooth angles, larger, but less saturated, variety of colors [95,96], not being common to find regions of constant color. Pixel to pixel color transitions have different models in natural and synthetic images. Despite these differences, it is possible to verify that the proposed approach presents better results when compared to some algorithms proposed by other authors.

### 5.1. Comparison with Colour-Texture Segmentation Algorithms

Over the years, several algorithms have been proposed in the area of image segmentation. In order to evaluate the performance and highlight the contribution of the proposed approach in the image segmentation field, side-by-side comparisons are presented between the results returned by the proposed system and the results returned by some algorithms proposed by other authors. To perform this comparison, we took as reference the work presented by Ilea et al. [97], where an evaluation and categorization of the most relevant algorithms for image segmentation are done based on the integration of colour–texture descriptors.

Table 2 identifies and briefly describes the algorithms considered in the comparison.

**Figure 26:**
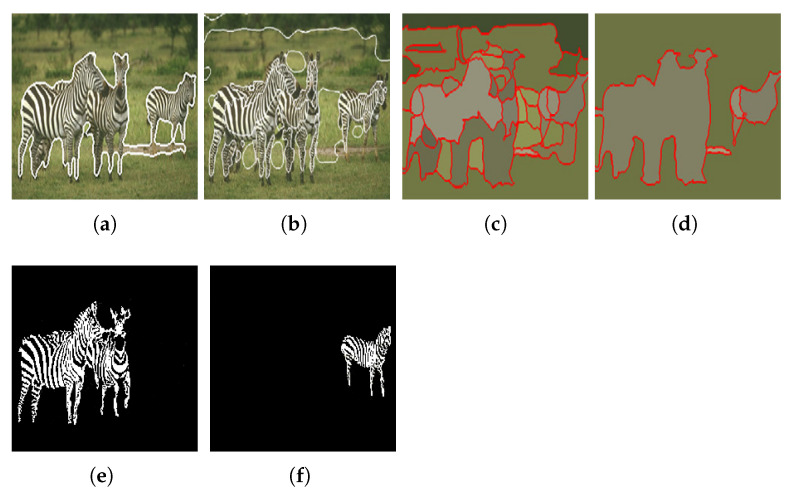
Results obtained using (**a**) Ilea and Whelan [105] algorithm; (**b**) Hoang et al. [98] algorithm [105]; (**c**) Yang et al. [100] algorithm when γ = 0.1; (**d**) Yang et al. [100] algorithm when γ = 0.2; (**e**,**f**) the proposed approach. Images (**a**–**d**) referenced from Ilea and Whelan [105].

Figure 26 shows the results obtained by the proposed approach and by the algorithms proposed by Ilea and Whelan [105], Hoang et al. [98], and Yang et al. [100].

When compared with the algorithms of Hoang et al. [98] and Yang et al. [100], it can be verified that the proposed approach presents a more accurate segmentation of the objects in the image. Regarding the algorithm of Ilea and Whelan [105], despite the segmentation accuracy being very similar, the approach we propose has the advantage of being able to separate the two objects of interest present in the image.

**Figure 27:**
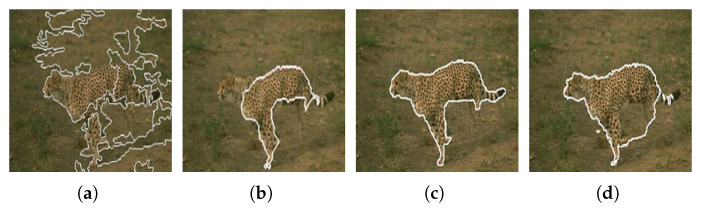
Results obtained using the algorithms proposed by (**a**) Deng and Manjunath [99]; (**b**) Chen et al. [101]; (**c**) Han et al. [102]; (**d**) Rother et al. [103]. Images referenced from Ilea and Whelan [105].

**Figure 28:**
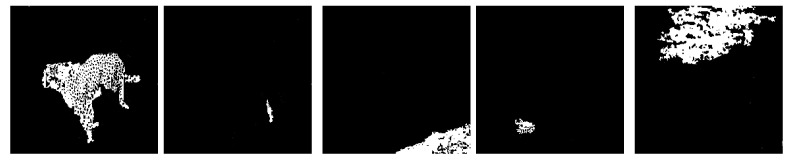
Objects extracted by the proposed approach.

Figure 27 and Figure 28 shows the results obtained by the proposed approach and by the algorithms proposed by Deng and Manjunath [99], Chen et al. [101], Han et al. [102] and Rother et al. [103].

With the analysis of the results presented in Figure 27 and Figure 28, we can observe that the proposed approach is more accurate than those proposed by the authors Deng and Manjunath [99], Chen et al. [101], Han et al. [102] and Rother et al. [103], extracting in a more complete way the object of interest present in the image.

**Figure 29:**
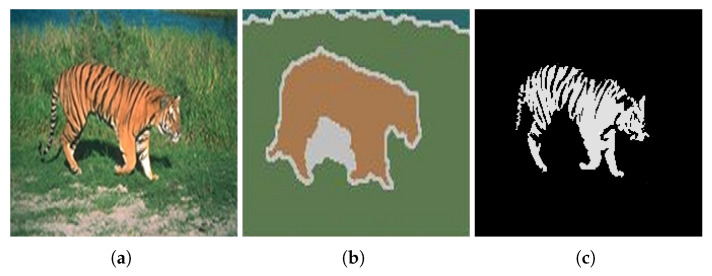
(**a**) Original image; (**b**) results obtained using the algorithm proposed by Carson et al. [104]; (**c**) results obtained using the algorithm proposed by the proposed approach. Images (**a**,**b**) referenced from Ilea and Whelan [105].

Figure 29 shows the results obtained by the proposed approach and by the algorithm proposed by Carson et al. [104].

Unlike the algorithm proposed by Carson et al. [104], the approach we propose has the ability to segment the tiger in the image, separating its tail from its body, thus achieving a more accurate result.

**Figure 30:**
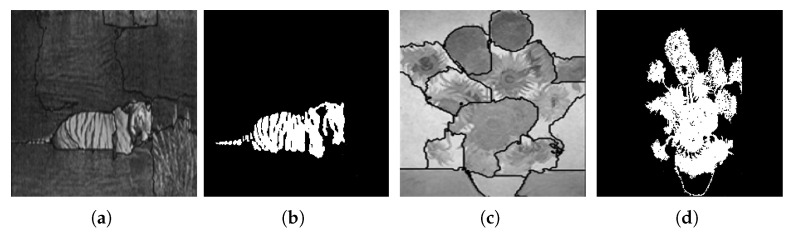
Results obtained using (**a**) the algorithm proposed by Malik et al. [69]; (**b**) the proposed approach; (**c**) the algorithm proposed by Malik et al. [69]; (**d**) the proposed approach. Images (**a**,**c**) referenced from Malik et al. [69].

Figure 30 shows the results obtained by the proposed approach and by the algorithm proposed by Malik et al. [69].

Finally, and when compared with the algorithm proposed by Malik et al. [69], it is possible to verify once again that the approach we propose is capable of performing the segmentation of regions of interest in a more accurate way. Note its accuracy in the segmentation of the image of the Sunflowers painting, where it is possible to differentiate the flowers from their stems.

### 5.2. Comparison with Deep Learning Approaches

When compared to a deep learning mechanism (object classification and detection), the approach proposed in this work presents the following main differences:

- Typical deep learning approaches require exhaustive training phases so that the model can correctly identify objects in an image. The results produced by the proposed system are achieved with only image processing techniques and require no training data.
- Deep Learning mechanisms are usually very limited regarding what they can identify in images, typically being capable of correctly identifying a small number of very general classes.
- Deep Learning mechanisms work much better with natural images and are mostly unbeatably accurate in real-life datasets. Building and training a network for graphical images (trademark images) requires a huge amount of data and would most likely never be as accurate as necessary, given the extreme variety of color composition, graphical styles, calligraphy and typography used in images.
- The system proposed in this work can be manually adjusted to each image’s complexity and graphical density, whereas deep learning models would require significant amounts of fine-tuning and retraining to achieve the same level of versatility.
- Unlabeled Detection: The proposed system is not prepared to identify a series of predetermined objects in images. Regions are proposed with regard to pixel blobs and distance measuring, meaning it is practically unlimited in regard to what it can extract.

## 6. Conclusions and Future Work

The main goal of this work was to develop an image region extraction system capable of overcoming the difficulties associated with having a highly diversified dataset of trademark graphic images. The proposed system consists of a multistage algorithm that takes an RGB image as input and produces multiple outputs, corresponding to the extracted regions. The proposed approach has a linear complexity, presenting runtimes compatible with real-time applications. The experiments performed investigated several different image segmentation techniques, including K-Means Clustering, watershed segmentation and connected component analysis to achieve the best possible results. The results obtained show adequate features of region extraction when processing graphic images from the test datasets, where the system correctly distinguishes the most relevant visual elements from the images with minimal adjustments, disregarding irrelevant information such as text. Notwithstanding other hybrid approaches that make use of one or more of the algorithms that integrate this system have been proposed, such proposals are not adequate in the context in which this work is inserted, as they do not allow the extraction of only the graphical objects considered relevant, disregarding irrelevant objects, such as the text. By comparing the results obtained by applying the proposed approach to natural images, with those presented by other authors, it is possible to verify that the developed system performs the segmentation of regions of interest of an image in a more accurate way. Although the main scope of the work presented is to integrate the proposed system in a series of image comparison systems, we aim to develop a model matching system that can identify the objects extracted by this approach in other images, as well as a review of image comparison metrics as a means of obtaining results comparable to those of graphical search engines.

## Figures and Tables

**Table 1:** Average algorithm execution time.

Dataset	Sample Size	Elapsed Time
National jurisdiction trademark images	19,102	2865 s
	1	0.1538 s
International jurisdiction trademark images	45,690	6542 s
	1	0.1542 s

**Table 2:** Approaches proposed by other authors with which the results obtained by the proposed approach were compared.

Colour-Texture Segmentation Algorithm	Summary
Hoang et al. [98]	The colour and texture information is included in the segmentation process. The RGB image is converted into a Gaussian colour model. Primary colour–texture features are extracted from each colour channel using a set of Gabor filters. Feature vectors, whose dimensionality is reduced by applying Principal Component Analysis, and used as inputs for a K-Means algorithm, providing initial segmentation that is refined by a region-merging procedure.
JSEG-Deng and Manjunath [99]	Consisting of two independent steps: color quantization and spatial segmentation. In the first step, image colors are quantized in different classes, which are used to create an image class map. The image segmentation results from the application of a region growing method to the set of multiscale images, formed through the application of the class map based segmentation evaluation criterion.
CTM-Yang et al. [100]	Colour–texture features at pixel level are extracted simultaneously by stacking the intensity values within a 7x7 window for each band of the CIE Lab converted image. Segmentation is formulated as a data clustering process. To reduce the dimensionality of the colour–texture vectors, Principal Component Analysis is used. To overcome the difficulty related to the fact that often the colour–texture information cannot be described with normal distributions, a coding-based clustering algorithm is employed that is able to accommodate input data defined by degenerate Gaussian mixtures.
Chen et al. [101]	Segmentation of natural images into perceptually distinct regions with application to content-based image retrieval. Local colour features are extracted using a spatially Adaptive Clustering Algorithm. Texture features are computed through a multi-scale frequency decomposition procedure. Colour and texture features are integrated using a region growing algorithm that generates a primary segmentation that is improved through a post-processing step that implements a border refinement procedure.
Han et al. [102]	A segmentation framework developed to identify the foreground object in natural colour images. Colour features are extracted from the CIE Lab converted colour image. Texture features are computed from the luminance component of the input image using the multi-scale nonlinear structure tensor. To reduce the dimensionality of the colour–texture feature space, the colour information is clustered using a binary tree quantisation procedure and the features in the texture domain are clustered using a K-Means algorithm. The resulting colour and texture features are modelled by Gaussian Mixture Models and integrated into a framework based on the GrabCut algorithm. The accuracy of the algorithm is improved by an adaptive feature integration strategy that consists of adjusting a weighting factor for colour and texture in the segmentation process.
GrabCut-Rother et al. [103]	A graph-cut approach extension, with a simpler user interaction and an iterative version of the optimization method. An algorithm for “border matting” is used to estimate simultaneously the alpha-matte around an object boundary and the colours of foreground pixels.
Blobworld-Carson et al. [104]	The goal of the proposal is to partition the input image in perceptual coherent regions. It includes an isotropy, polarity and contrast features in a multi-scale texture model. Colour features are extracted on an independent channel from the CIE Lab converted image previously filtered with a Gaussian operator. For automatic colour–texture image segmentation, the distribution of the colour, texture and position features are jointly modeled using Gaussian Mixture Models. The Blobworld algorithm is able to segment the image into compact regions, being suitable to integrate a content-based image retrieval system.
CTex-Ilea and Whelan [105]	Colour and texture are treated on separate channels. Colour segmentation involves the statistical analysis of data using multi-space colour representations. After filtering the input data using a Gradient-Boosted Forward and Backward anisotropic diffusion algorithm, the colour segmentation algorithm extracts the dominant colours and identifies the optimal number of clusters using an unsupervised procedure based on a Self Organising Map network. After, the image is analysed in a complementary colour space where the number of clusters previously calculated performs the synchronisation between the two computational streams of the algorithm. Finally, clustered results obtained for each colour space form the input for a multi-space clustering process that outputs the final colour segmented image. The extraction of the texture features from the luminance component of the original image uses a multi-channel texture decomposition technique based on Gabor filters. The colour and texture features are integrated in an Adaptive Spatial K-Means framework that partitions the data mapped into the colour-texture space by adaptively sampling the local texture continuity and the local colour smoothness in the image.
Malik et al. [69]	An algorithm for partitioning grayscale images into disjoint regions of coherent brightness and texture, where cues of colors and texture differences of natural images are exploited simultaneously. Contours are treated in the intervening contour framework, while texture is analysed using textons. Given the different domain of applicability of each cue, a gating operator is introduced based on the texturedness of the neighbourhood at a pixel. Given a local measure of the similarity between nearby pixels, the spectral graph theoretic framework of normalized cuts is used to find partitions of the image in regions of coherent texture and brightness.

## Data Availability

The data presented in this study are available on request from the corresponding author. The data are not publicly available due to company privacy matters; however, all data contained in the dataset mentioned in Section 4.1 are publicly available.

## Abbreviations

The following abbreviations are used in this manuscript:

CCA	Connected Component Analysis
CBIR	Content Based Image Retrieval
CNN	Convolutional Neural Network
R-CNN	Region-Based Convolutional Neural Network
OCR	Optical Character Recognition
